# Ferritin heavy chain is a negative regulator of ovarian cancer stem cell expansion and epithelial to mesenchymal transition

**DOI:** 10.18632/oncotarget.11495

**Published:** 2016-08-22

**Authors:** Nadia Lobello, Flavia Biamonte, Maria Elena Pisanu, Maria Concetta Faniello, Žiga Jakopin, Emanuela Chiarella, Emilia Dora Giovannone, Rita Mancini, Gennaro Ciliberto, Giovanni Cuda, Francesco Costanzo

**Affiliations:** ^1^ Centro di Ricerca di Biochimica e Biologia Molecolare Avanzata, Dipartimento di Medicina Sperimentale e Clinica, Università degli Studi “Magna Graecia”, Catanzaro, Italy; ^2^ Dipartimento di Medicina Clinica e Molecolare, Sapienza Università di Roma, Italy; ^3^ Laboratorio di Biologia Cellulare e Molecolare, Dipartimento di Chirurgia “P. Valdoni”, Sapienza Università di Roma, Italy; ^4^ Faculty of Pharmacy, University of Ljubljana, Slovenia; ^5^ Dipartimento di Medicina Sperimentale e Clinica, Università degli Studi “Magna Graecia”, Catanzaro, Italy; ^6^ Centro Interdipartimentale di Servizi e Ricerca, Università degli Studi “Magna Graecia”, Catanzaro, Italy; ^7^ Istituto Nazionale per lo Studio e la Cura dei Tumori “Fondazione G. Pascale”, Napoli, Italy

**Keywords:** ferritin heavy chain, ovarian cancer, cancer stem cells, EMT, miRNAs

## Abstract

**Objectives:**

Ferritin is the major intracellular iron storage protein essential for maintaining the cellular redox status. In recent years ferritin heavy chain (FHC) has been shown to be involved also in the control of cancer cell growth. Analysis of public microarray databases in ovarian cancer revealed a correlation between low FHC expression levels and shorter survival. To better understand the role of FHC in cancer, we have silenced the FHC gene in SKOV3 cells.

**Results:**

FHC-KO significantly enhanced cell viability and induced a more aggressive behaviour. FHC-silenced cells showed increased ability to form 3D spheroids and enhanced expression of NANOG, OCT4, ALDH and Vimentin. These features were accompanied by augmented expression of SCD1, a major lipid metabolism enzyme. FHC apparently orchestrates part of these changes by regulating a network of miRNAs.

**Methods:**

FHC-silenced and control shScr SKOV3 cells were monitored for changes in proliferation, migration, ability to propagate as 3D spheroids and for the expression of stem cell and epithelial-to-mesenchymal-transition (EMT) markers. The expression of three miRNAs relevant to spheroid formation or EMT was assessed by q-PCR.

**Conclusions:**

In this paper we uncover a new function of FHC in the control of cancer stem cells.

## INTRODUCTION

Iron uptake, storage and utilization are among the most highly regulated biochemical pathways in the cell. Iron is essential for energy metabolism, DNA synthesis and oxygen transport but is also able to catalyse the generation of reactive oxygen species (ROS) through Fenton chemistry, which, in turn, causes lipid and protein peroxidation and DNA breakage [1]. Ferritin, a multimeric globular protein of approximately 450 kDa, stores the intracellular iron in a non-toxic and readily available form and is therefore essential in maintaining the cellular redox status [2]. Its role as major antioxidant molecule has been long recognized in physiologic and pathologic conditions, such as apoptosis [3], inflammation [4, 5], vascular protection and neurodegeneration [6, 7].

In eukaryotes, ferritin is localized in cytoplasm, mitochondria and nucleus. The cytoplasmic ferritin is constituted by 24 subunits of heavy- (H; FHC; FTH) and light-type (L; FTL) assembled in a nano-cage with a central cavity where up to 4500 atoms of iron can be stored [1]. The L subunit is absent in nuclear form and mitochondrial ferritin; in the latter, the shell is composed by a single subunit with a 75% sequence identity to the H ferritin [8, 9]. Two different genes, both belonging to complex multigene families and subjected to different transcriptional control mechanisms, code the heavy- and light-subunits, while an intronless H-type gene codes the mitochondrial form [10, 11]. Despite an extensive homology in the aminoacid sequence, FHC and FTL perform different functions: FHC has a ferroxidase activity and is devoted to rapid iron uptake and release, while FTL contributes to long-term iron storage [11]. Recently, it has been shown that FHC, aside from its role in iron metabolism, is involved in many cell regulatory pathways such as cell proliferation [9], chemokine signalling [12], and angiogenesis [13]. In at least two cases, the regulatory function of FHC is due to its ability to physically interact with other molecules: FHC can bind to, and transcriptionally activate, under oxidative stress conditions, the p53 protein [14]. Analogously, FHC may also interact with the chemokine receptor CXCR4, down-regulating the signal transduction pathway triggered by its ligand, CXCL12. The CXCR4 receptor is highly expressed in a variety of human tumors: the ability of FHC to modulate the CXCR4 pathway might partly explain the complex role that FHC plays in the process of neoplastic transformation [12].

FHC transcription is modulated, either positively or negatively, by several cancer-related genes, such as p53 [15], E1A [16], cMyc [17], cJun [18]. Furthermore, it appears that pathways related to inflammation may also regulate FHC expression, since its transcription is activated by TNFα and interleukin-1α (IL-1α) [19]. We have recently demonstrated that FHC down-modulation by shRNA interference strongly reduces, both *in vivo* and *in vitro*, melanoma cell proliferation [20], while, in the erythroleukemia K562 cell line, it modulates the expression of a specific set of onco-miRNAs [21].

According to the cancer stem cell hypothesis, cancer is maintained by a subpopulation of cells with stem cell like properties (CSCs), with extensive capacity of self-renewal [22]. Putative CSCs are mostly quiescent for proliferation, are resistant to chemotherapeutic agents and therefore are believed to be involved in disease relapse. The identification of factors which regulate CSC survival and their expansion, is believed to open up new avenues for therapeutic intervention. The identification of CSCs through the use of surface markers has been the subject of intensive studies with controversial interpretations. As a matter of fact, nowadays the general consensus is that there is no universal surface marker for CSCs of a given origin, although some markers appear more frequently associated with a CSC functional phenotype [23, 24]. Among them, ALDH1A1 expression and its enzymatic activity seem to correlate well with the presence of CSCs. ALDH1A1 has been associated with metastasis, drug resistance, and a poor prognosis in several tumors [25]. An alternative and highly practical approach to the enrichment of putative CSCs is the *in vitro* 3D spheroid propagation assay [26]. This is based on the evidence that terminally differentiated cancer cells, when cultured in low attachment plates and in a medium without serum supplemented with EGF and bFGF, undergo anoikis [27]. In contrast, CSCs grown in the same culture conditions are resistant to anoikis and when seeded at low density upon repeated cell divisions tend to form 3D spheroids. We and others have used therefore the 3D spheroid propagation assay to quantify CSCs in a given cell population, identify genes preferentially expressed in spheroids and demonstrate their role in CSC maintenance and expansion [28–31].

In this paper, using a human cancer cell line SKOV3, we unexpectedly discover a new role for FHC as a repressor of cancer proliferation and, most importantly, CSC propagation. Through a series of assays, we propose that this new function of FHC is, at least in part, exerted through the regulation of a subset of miRNAs involved in cell migration and control of epithelial to mesenchymal transition.

## RESULTS

### Low FHC expression is linked to poor prognosis in ovarian cancer

**Figure 1:**
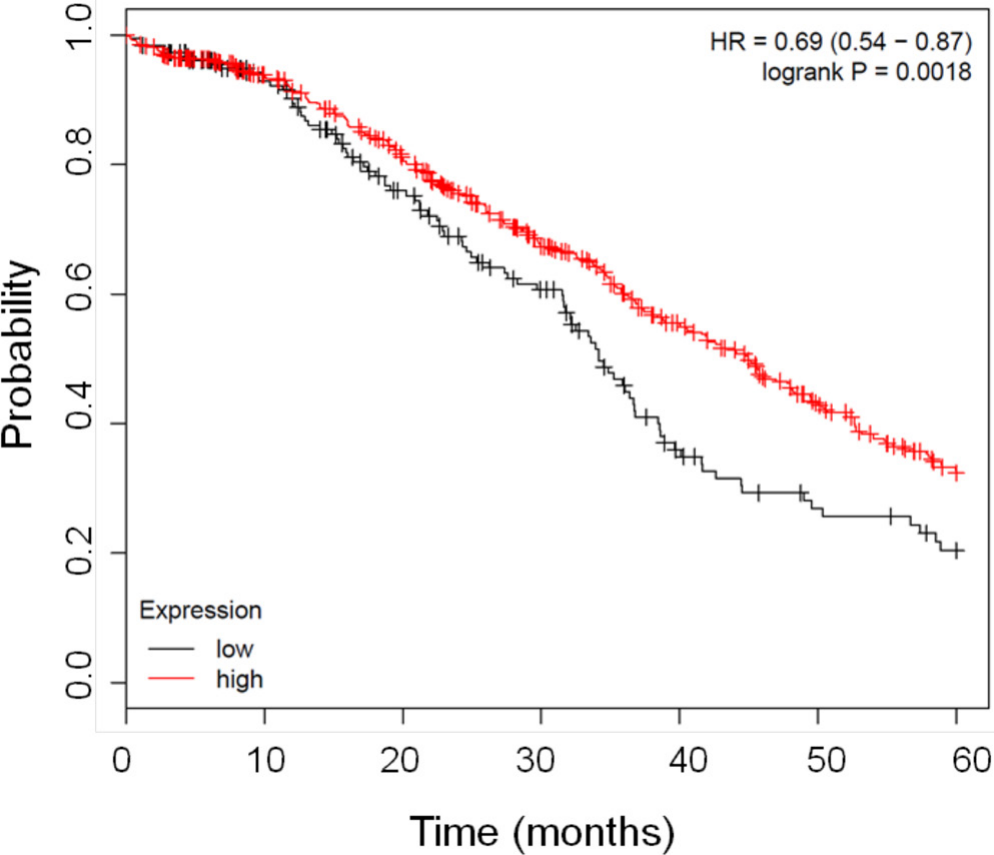
Kaplan Meier curves showing the good prognostic effect on overall survival of the higher expression of FHC gene The graph shows the correlation between overall survival and FHC expression in patients affected by ovarian cancer. The red line represents samples with higher FHC expression *n* = 540 while black line indicates patients with lower FHC expression *n* = 183. The Kaplan–Meier survival plot was generated by www.kmplot.com/ovar [32].

In order to assess the prognostic relevance of FHC gene expression in ovarian cancer we interrogated published ovarian cancer microarray datasets using available online tools (www.kmplot.com/ovar). To this purpose we combined together multiple large microarray data sets obtained from GEO and TCGA databases [32]. Patients were filtered using stage, histology, grade. We selected patients with serous ovarian cancer at stage II-III, grade 3. Samples were divided into 2 groups according to the median FHC expression, having high and low FHC expression respectively. In Figure 1 is a Kaplan Meyer representation of the results. We found that patients with lower FHC mRNA expression have a statistically significant shorter survival (*p* = 0.0018). These data led us to hypothesize that high FHC expression may be associated with a less aggressive disease.

### SKOV3 cells silenced for FHC have a more aggressive tumorigenic phenotype

In order to better dissect the role of FHC, the cancer cell line SKOV3 was subjected to targeted knock down of FHC gene expression via shRNA silencing (see Materials and Methods). Supplementary Figure S1 shows that this approach was successful both at RNA (panel A and B) and protein (panel C) levels. Endogenous FHC protein and RNA levels were decreased at least 10-fold. Immunofluorescence microscopy confirmed qRT-PCR and Western blot findings (panel D).

**Figure 2:**
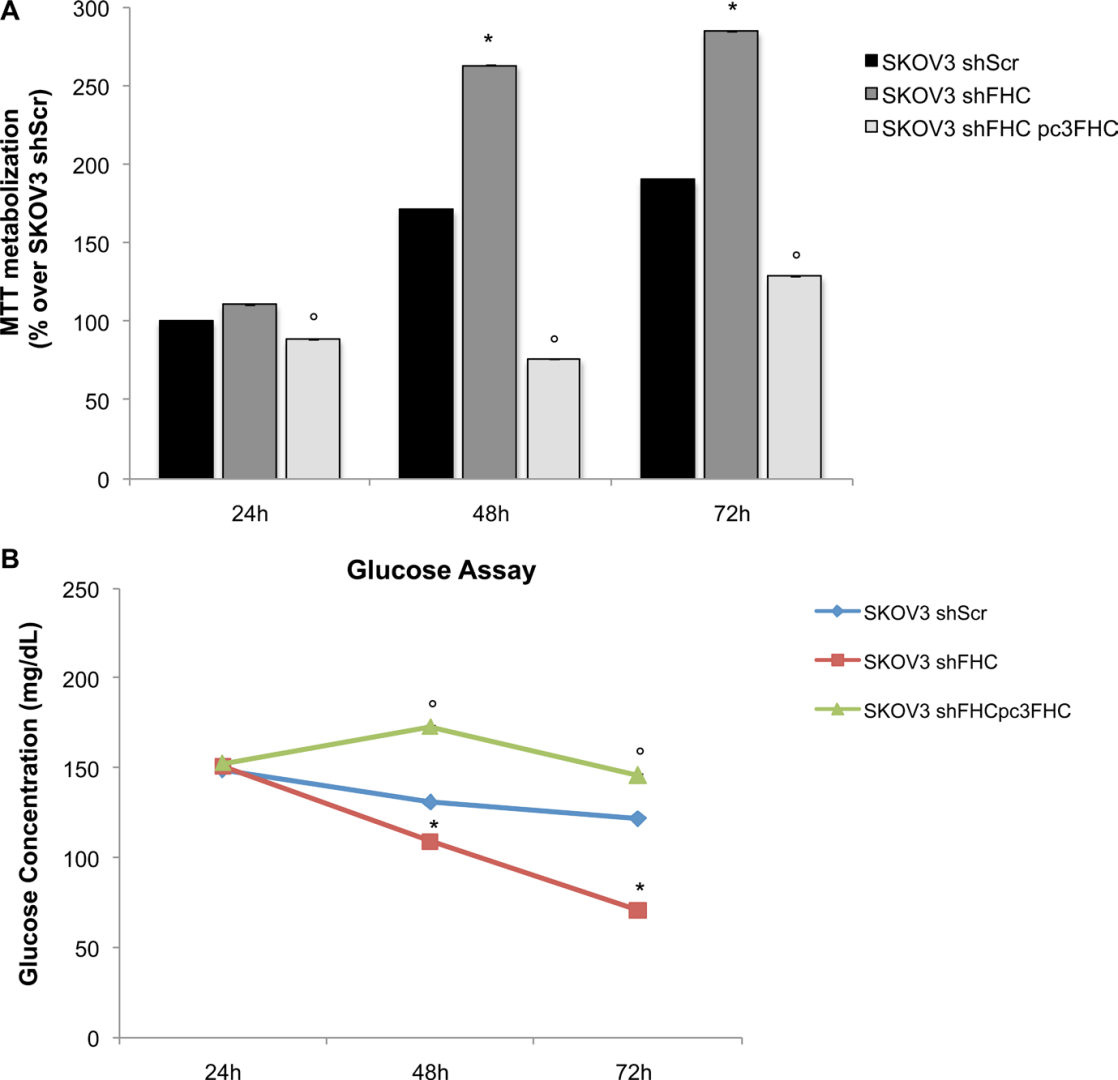
FHC-silencing confers a more malignant phenotype through an increase in cellular proliferation ability and glucose uptake Cell proliferation and viability, measured by MTT assay, is higher in FHC-silenced compared to non-silenced SKOV3 cells at 24, 48 and 72 h. FHC reconstitution leads to a significant reduction of SKOV3 shFHC cell proliferation at each time point. All the experiments were performed in triplicate (data are represented as mean +/− SD) and the values are given as % of MTT metabolization over the cell type used as control. *p value ≤ 0.05 compared with SKOV3 shScr; °*p* value ≤ 0.05 compared with SKOV3 shFHC (**A**). FHC-silenced SKOV3 cells exhibit significant increased glucose uptake than SKOV3 control cells. While at 24 h no differences in glucose concentration in culture media are detectable, at 48 and 72 hours glucose concentration is significantly lower in SKOV3 shFHC compared to SKOV3 shScr cells. *p value ≤ 0.05. When FHC expression is restored, glucose SKOV3 cells exhibit a significant reduced glucose uptake at 48 and 72 hours. °*p* value ≤ 0.05. The experiment was performed in triplicate and data are represented as mean +/− SD (**B**).

We and others have demonstrated that FHC silencing may modulate in different ways the tumorigenic phenotype of several cell lines [20, 21]. We first assessed if lack of FHC expression causes changes in the proliferation rate of SKOV3 shFHC *vs* control SKOV3 shScr cells using a colorimetric methyl-thiazolyl-tetrazolium (MTT) assay. The results, shown in Figure 2A, demonstrate that FHC silencing increased cell proliferation significantly at 48 and 72 hours.

In order to investigate the effects of FHC functional suppression on the metabolism of SKOV3 cells, glucose concentration was determined in the cell culture medium of SKOV3 shFHC cells *vs* control SKOV3 shScr cells. We observed (Figure 2B) that loss of FHC led to a significant decrease of glucose in the cell medium in a time-dependent manner, thus suggesting that SKOV3 shFHC cells have an accelerated metabolism and are indeed consuming a significantly higher amount of the major energy-producing source.

In order to rule out potential off-target effects of the shRNA used for FHC silencing, ferritin heavy chain expression was reconstituted in SKOV3 shFHC cells (SKOV3 shFHC^pc3FHC^) by transfecting an expression vector for the full length human FHC cDNA modified not to be targeted by the shRNA used for silencing (Supplementary Figure S2). As expected, FHC re-expression in silenced SKOV3 shFHC cells reduced the proliferation rate back to initial levels and reduced glucose metabolism (Figure 2A and 2B).

**Figure 3:**
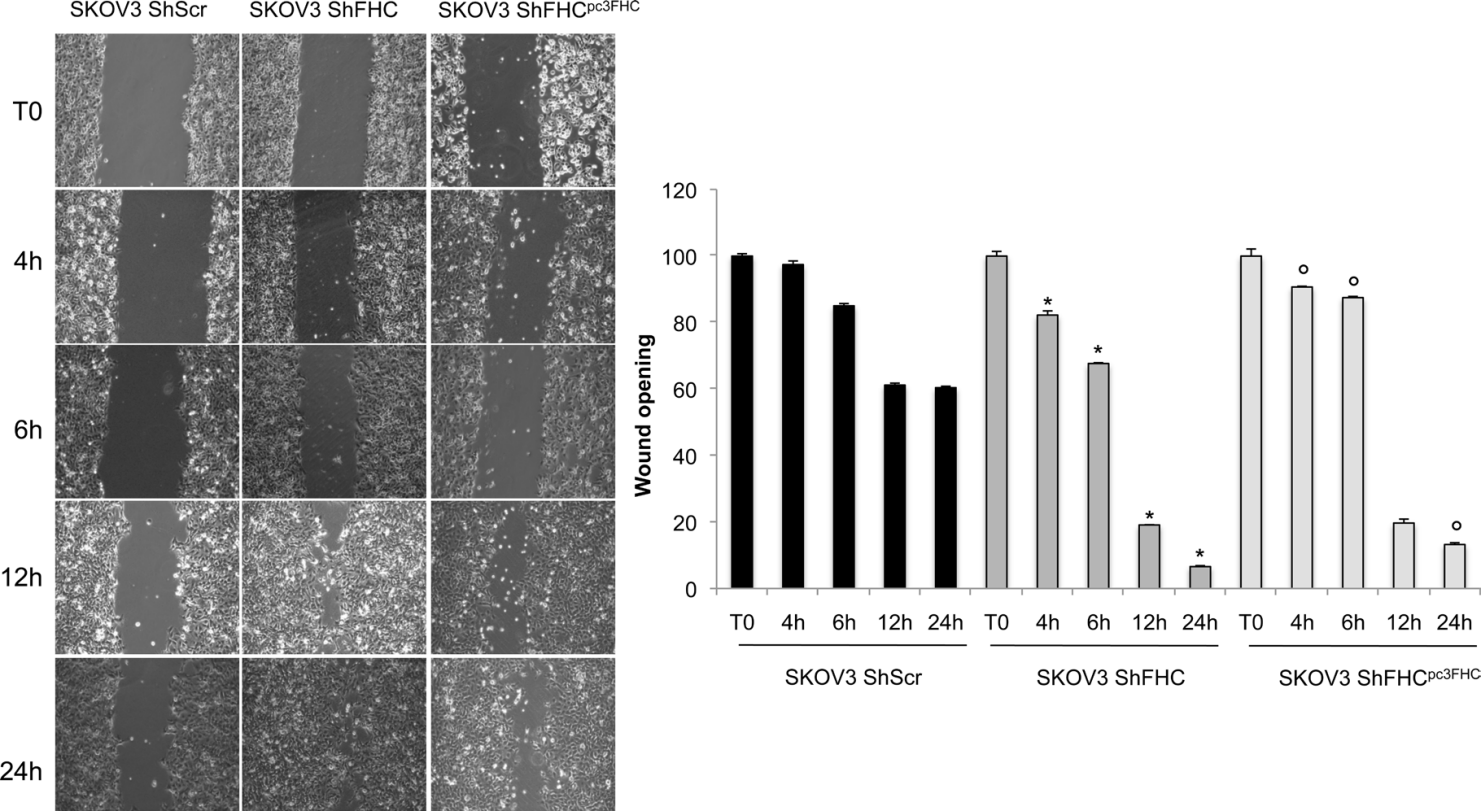
FHC-silencing increases cell migration ability Wound healing assay was conducted to measure migration capacity of control (SKOV3 shScr), FHC-silenced (SKOV3 shFHC) and FHC-restored (SKOV3 shFHC^pc3FHC^) cells. FHC-silencing promotes cellular migration at 4, 6, 12 and 24 h. This ability is reduced by transiently FHC-reconsitution. Wound size was quantified by ImageJ 64 software. Images and quantification are representative of three different experiments and the values are given as mean +/− SD. *p value ≤ 0.05 compared with SKOV3 shScr. °*p* value ≤ 0.05 compared with shFHC.

Finally, we compared the migratory ability of SKOV3 shFHC *vs* control SKOV3 shScr cells in linear scratch assays on monolayer-grown cells. The results (Figure 3) show that the wound closure per field was significantly enhanced, also at short times, in FHC-lacking SKOV3 cells with respect to the shScr counterpart. FHC re-expression in FHC silenced cells was able to reduce only partially cell migration back to the same level of SKOV3 shScr control cells.

In order to confirm the previous observations we silenced FHC transiently in SKOV3 cells using a siRNA complementary to a different region of the FHC mRNA. The results (Supplementary Figure S3A and S3B), show also in this case increased proliferation measured with the MTT assays as well as increased migration.

Taken together, the functional assays reported above suggest that the product of the ferritin heavy chain gene exerts an important control of ovarian cancer SKOV3 cells progression *in vitro*, because its abrogation confers a more aggressive phenotype.

### Epithelial-to-mesenchymal transition and spheroid formation are promoted by FHC silencing

**Figure 4:**
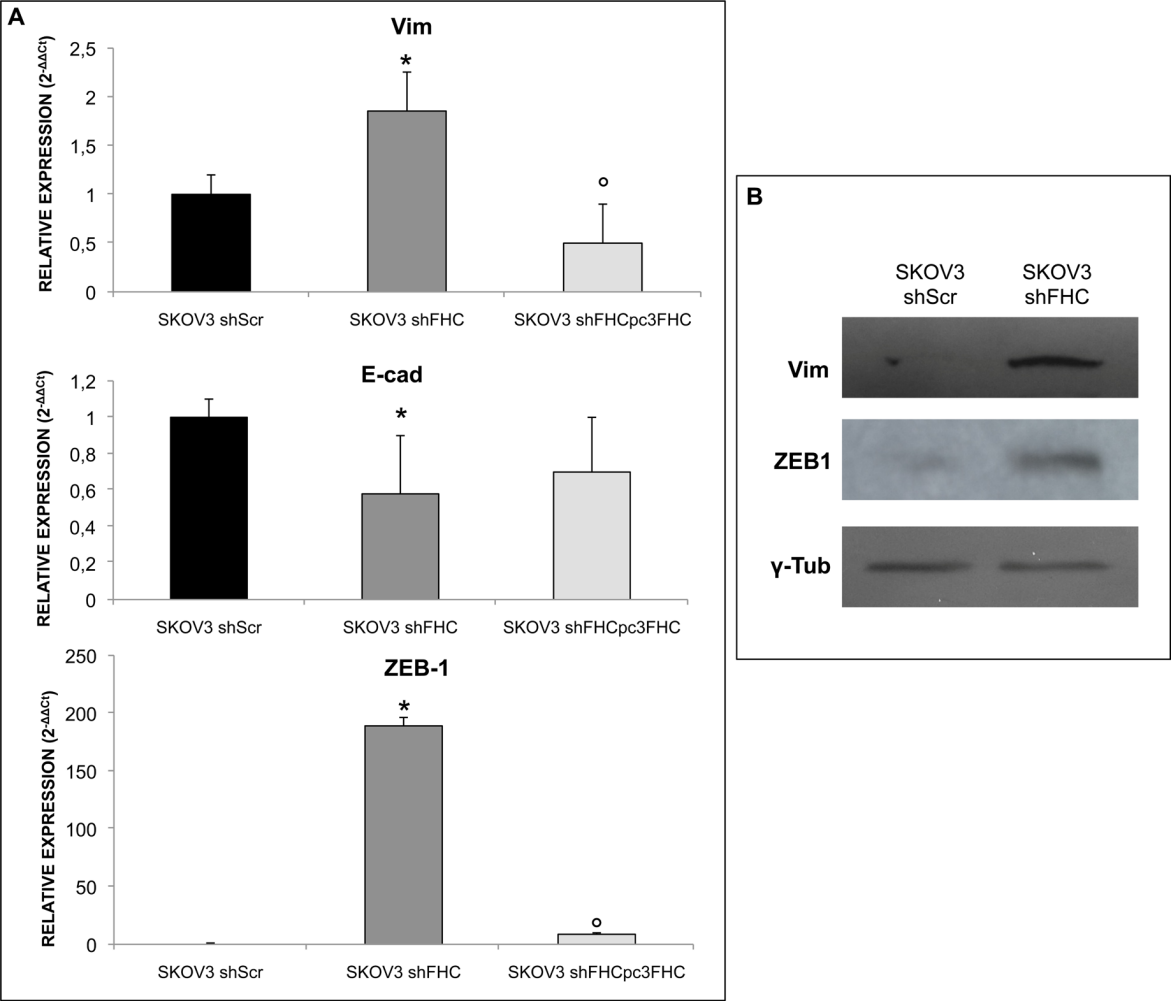
FHC silencing induces EMT in SKOV3 cells mRNA (**A**) and protein (**B**) expression levels of EMT markers: ZEB1, Vimentin and E-cadherin. ZEB1 is significantly over-expressed in SKOV3 shFHC compared to scrambled SKOV3 control cells at both mRNA and protein levels. The expression of Vimentin and E-cadherin, a mesenchymal and epithelial marker respectively, are inversely correlated. FHC-silencing significantly decreases epithelial marker expression while increasing the mesenchymal one. FHC-reconstitution significantly decreases Vimentin and ZEB-1 mRNA expression levels and increases, albeit without reaching a statistical significance, E-cadherin mRNA expression levels. The experiments were performed in triplicate. qPCR results are shown as mean +/− SD. *p value ≤ 0.05 compared with SKOV3 shScr. °*p* value ≤ 0.05 compared with shFHC.

Ovarian cancer is known to metastasize by releasing cancer cells in the peritoneum and dispersing them through the peritoneal fluid [33]. Epithelial to Mesenchymal Transition (EMT) plays a crucial role in the tumor invasion process through the disruption of cell-cell contacts, loss of apico-basal polarity, matrix remodelling, and increase motility and invasiveness [34]. We have monitored the expression of some EMT markers. To this end, Zinc Finger E-Box Binding Homeobox 1 (ZEB1), Vimentin and E-cadherin levels were first measured in SKOV3 shScr and in SKOV3 shFHC cells. ZEB1 is an important regulator of EMT and is a target of miR-150 [35]; Vimentin and E-cadherin, widely used indicators of stromal invasion by cancer cells, are strongly up- and down-regulated, respectively, during EMT [34]. Remarkably, upon silencing of FHC, SKOV3 cells showed a significant increase of ZEB1 and Vimentin levels both at the mRNA and protein levels, paralleled by a decrease of E-cadherin (Figure 4A and 4B), which suggests that metastasis and invasiveness might be kept under check by intracellular ferritin levels. This effect was specific because FHC re-expression in SKOV3 shFHC cells was able to revert the EMT markers phenotype induced by ferritin heavy chain silencing (Figure 4A). In agreement with previous findings, transient silencing of FHC with a siRNA mapping to a different region of FHC mRNA caused a significant increase in Vimentin and ZEB1 expression (Supplementary Figure S3C).

**Figure 5:**
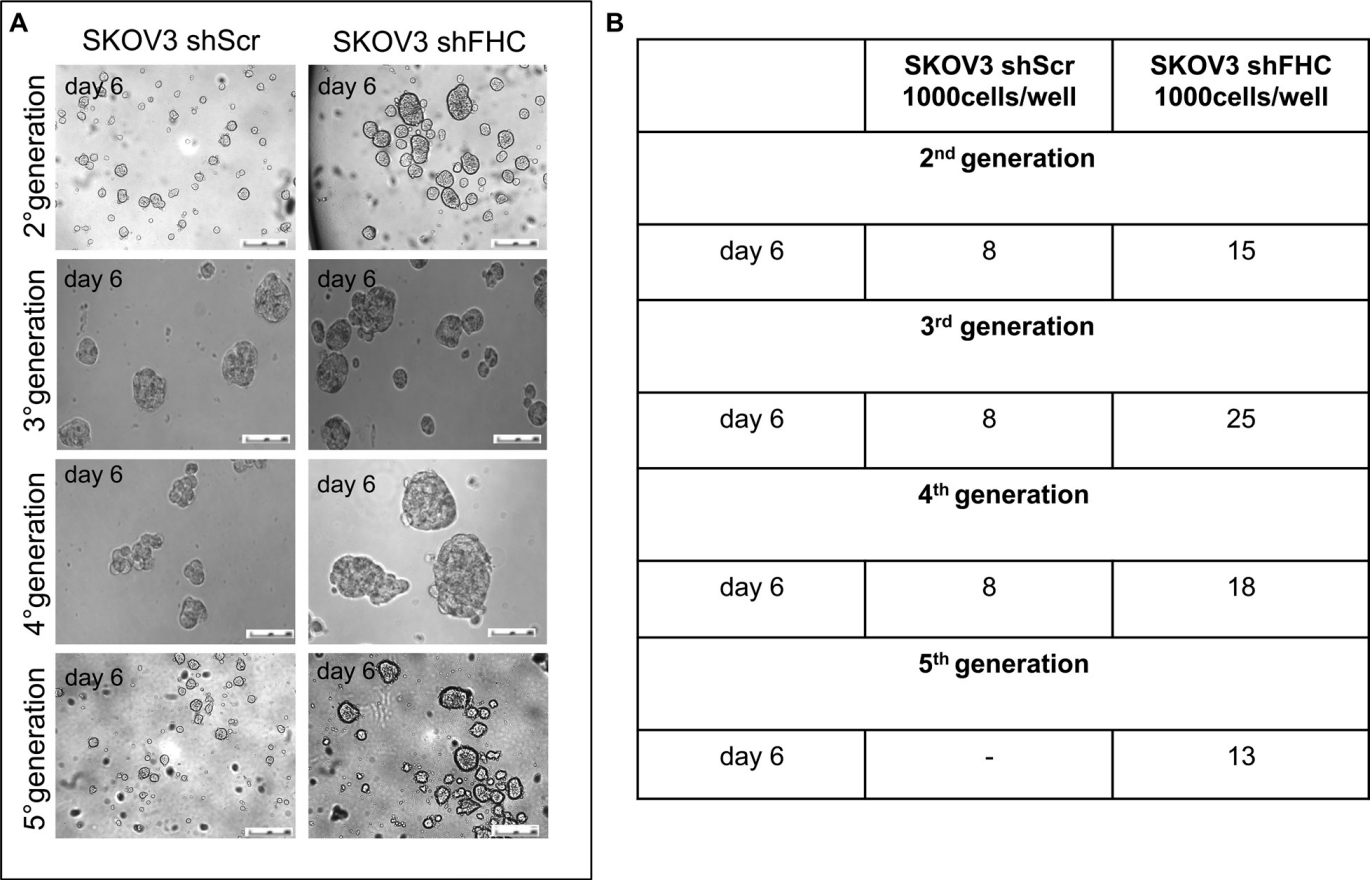
Enhanced spheroidogenesis ability of FHC-silenced SKOV3 cells SKOV3 shScr and SKOV3 shFHC cells were cultured in low-attachment conditions and formation of spheroids was observed. Photographs of representative spheroids from each cell type (**A**). Tumour spheroid number, counted at day 6 after each propagation. Results are reported as the average of 4 different fields (**B**). FHC-silencing induced a significant increase in spheroids number and size at each generation. Results are representative of three different experiments.

Spheroid formation, i.e. the generation of three dimensional cellular aggregates under non-adherent conditions, is a powerful *in vitro* assay to assess the presence of cancer initiating cells. Spheroid cultures over-expressing markers of stemness are more resistant to chemotherapy and are able to switch from an epithelial to a mesenchymal phenotype [28–30]. We carried out serial propagation of 3D spheroids both for SKOV3 shScr and shFHC in parallel and quantified spheroid numbers. The results shown in Panels A and B of Figure 5 demonstrate that knock down of FHC expression coincides with the formation of more rapid, abundant and larger tumor spheroids which look very often well encapsulated. Remarkably, analogous results were found when SKOV3 shScr and shFHC cells were plated at a very low density (100 cells/well) (Supplementary Figure S4).

**Figure 6:**
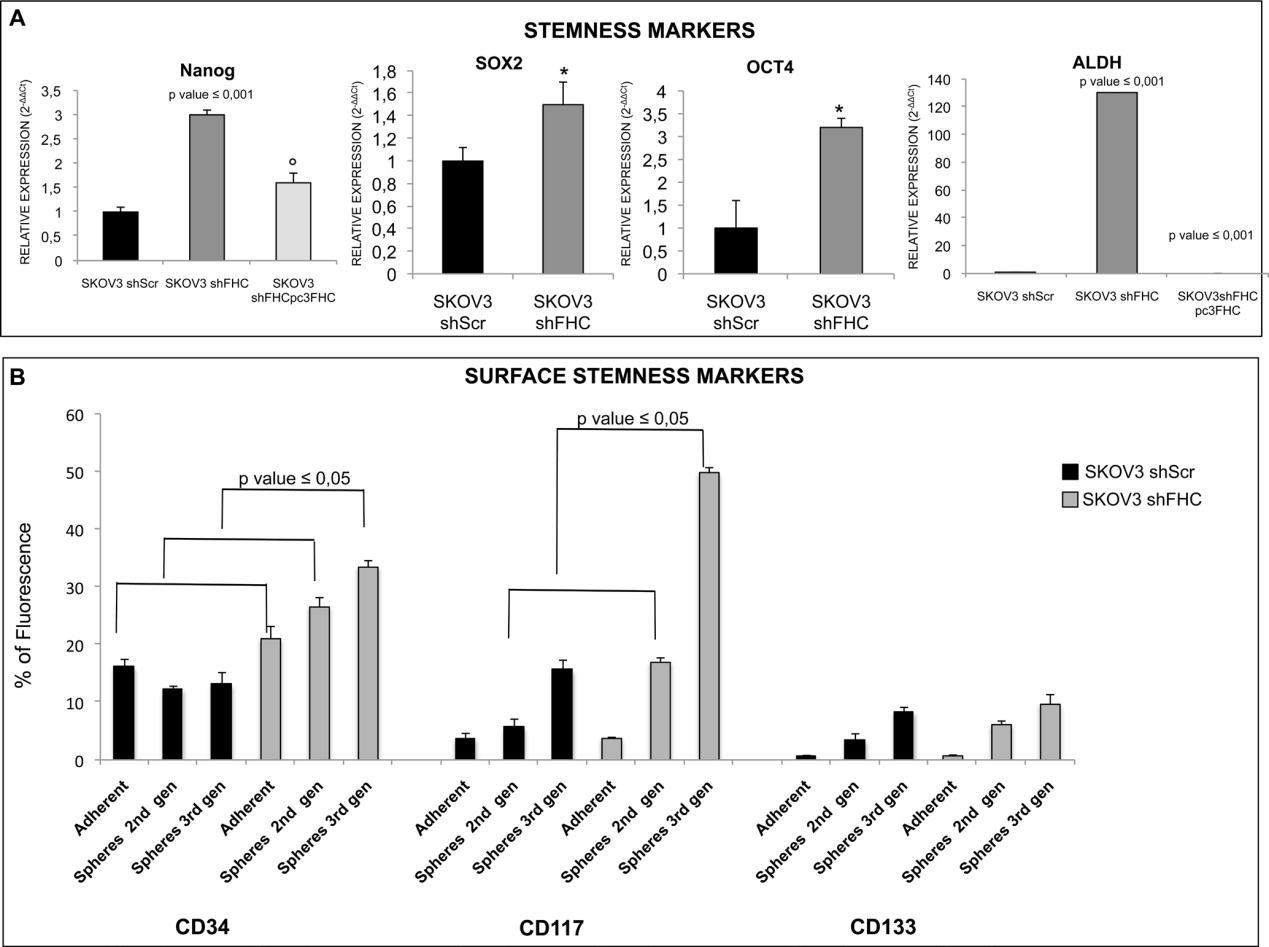
FHC-silencing promotes stemness in SKOV3 cells mRNA expression of stemness markers was evaluated by qRT-PCR. NANOG, SOX2, OCT4 expression increase with statistical significance in SKOV3 silenced cells compared to non-silenced SKOV3 cells. ALDH expression, in particular, is significantly higher in SKOV3 cells after FHC-silencing compared to scrambled SKOV3 control cells. Nanog and ALDH mRNA expression levels are significantly down-regulated after FHC-reconstitution in FHC-silenced cells.*p value ≤ 0.05 compared with SKOV3 shScr. °*p* value ≤ 0.05 compared with shFHC. qRT-PCR results are shown as means +/− SD (**A**). Surface stemness markers were evaluated by Flow cytometry analysis. CD34, CD117 and CD133 surface stemness markers are up-regulated in SKOV3 silenced cells compared to non-silenced cells. Only CD34 is significantly up-regulated in SKOV3 shFHC compared to SKOV3 shScr either in adherent or spheroid conditions; CD117 is significantly up-regulated in FHC-silenced spheroids compared to non-silenced spheroids at second and third generations while no statistical significance is observed for CD133 differential expression. The experiment was performed in triplicate and data are shown as mean +/− SD (**B**).

These findings suggest that the population of SKOV3 cells silenced for FHC may contain higher amounts of cancer stem cells. In order to confirm this hypothesis, we measured the expression level of several stem cell markers. The results show that spheroids formed by shFHC-SKOV3 express significantly higher levels of SOX2, Nanog, Oct4, ALDH, CD34, CD117, with the only exception of CD133 (Figure 6A and 6B). Again, also this effect was specific because FHC re-expression in SKOV3 shFHC cells was able to fully revert the effect of ferritin heavy chain silencing (Figure 6A). When transient FHC knock down was carried out with a siRNA mapping at a different location, similar changes in stem cell marker expression were observed (Supplementary Figure S3C).

Remarkably, the inverse relationship between FHC expression and spheroid formation ability was not restricted to this ovarian cancer cell line because, when we silenced FHC in the human breast adenocarcinoma cell line MCF-7, similar changes were observed (Supplementary Figure S5).

### FHC silencing in SKOV3 cells modulates a subset of microRNAs involved in cancer cell migration and spheroid formation

The results presented in the previous section point to a prominent role of ferritin heavy chain in the control of ovarian cancer growth, in large part through inhibition of EMT and cancer stem cells propagation. In order to better understand the mechanism through which FHC exerts these functions, we started to analyze the expression level of selected microRNAs.

MicroRNAs are widely recognized as major modulators of gene expression programs. Among them, microRNA 125b (miR-125b) has been found playing a role in carcinogenesis, through several mechanisms, including promotion of neo-angiogenesis, induction of resistance to chemotherapy and inhibition of apoptosis [36]. Conversely, other reports suggest that miR-125b may reduce tumor cell migration and invasion [37]. We have recently described a significant increase of miR-125b expression in K562 erythroleukemia cells following FHC gene silencing [21].

**Figure 7:**
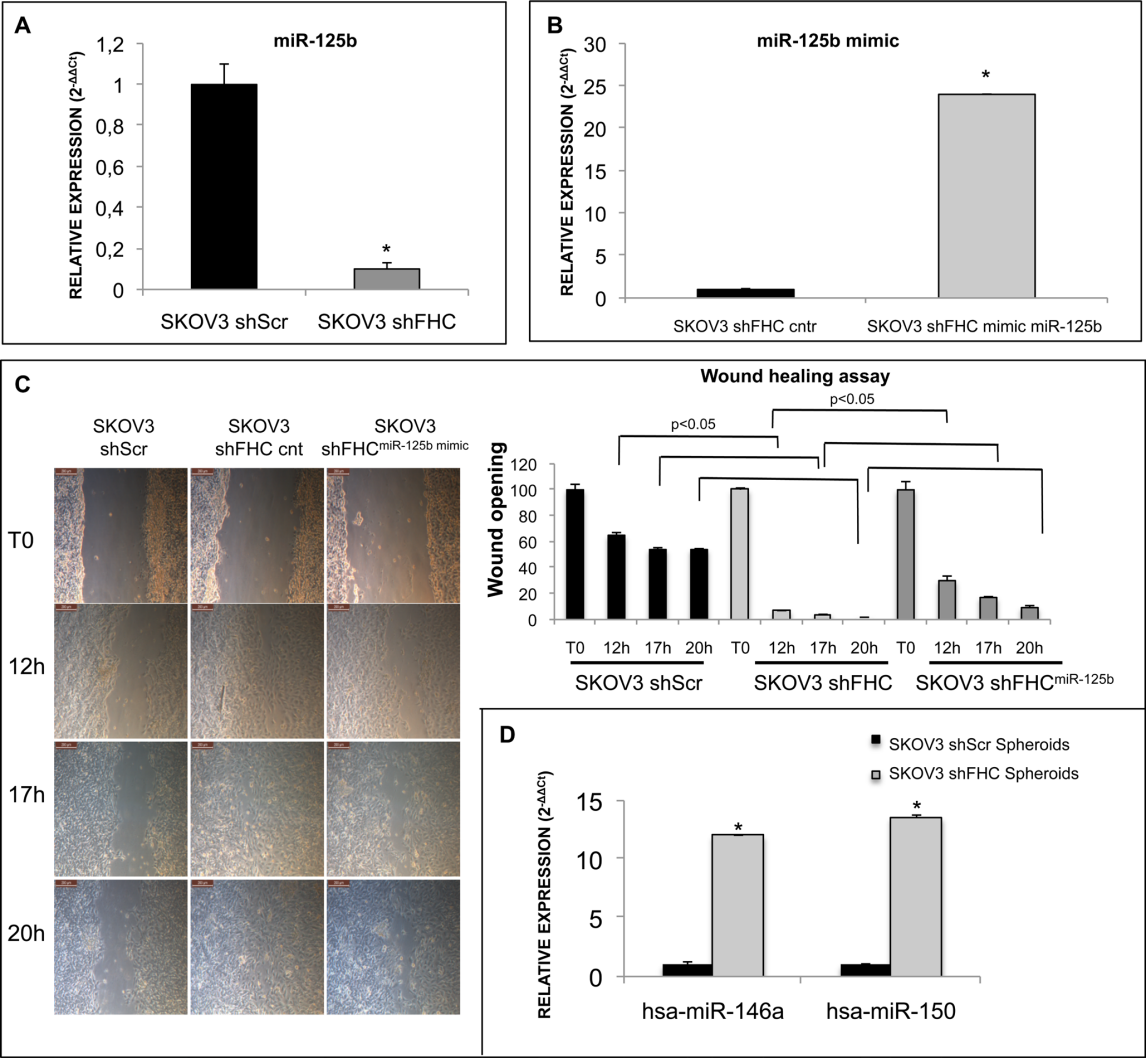
FHC intracellular amount affect migration ability of SKOV3 cells via modulation of miR-125b expression miR-125b expression level, measured by TaqMan Assay, is significantly reduced after FHC-silencing in SKOV3 cells (**A**). miR-125b mimic and scrambled control were transfected into SKOV3 shFHC cells. Cells transfected with miR-125b showed a higher level of miR-125b expression compared to scrambled control (**B**). Images of wound healing assay in shScr, shFHC and shFHC^miR-125b mimic^ SKOV3 cells. Transient over-expression of miR-125b inhibited the migration of FHC-silenced SKOV3 cells at 12, 17 and 20 h. The wound opening was quantified with ImageJ 64 software (**C**). miR-146a and miR-150, detected by TaqMan assay, are up-regulated in the second generation of SKOV3 shFHC spheroids compared to controls (**D**). All data shown are the mean +/− SD of two different experiments. *p value ≤ 0.05.

Therefore, we measured miR-125b levels in shScr control and shFHC SKOV3 cells and found that the loss of FHC dramatically reduced miR-125b expression (Figure 7A). Apparently, miR-125b is downstream to FHC because when we restored its expression, miR-125b levels were totally rescued (Supplementary Figure S6). Finally, when we forced miR-125b expression in SKOV3 shFHC cells using a specific miRNA mimic (Figure 7B), cells showed only a partial impairment of cell migration (Figure 7C).

miR-150 and miR-146a have been recently linked to the formation of 3D spheroids in metastatic ovarian cancer. Experimental findings performed on proliferating OVCAR-8 and SKOV3 cells clearly indicated that both these microRNAs are able to promote spheroid formation [38]. We have tested, therefore, if the expression levels of miR-150 and miR-146a are modulated in FHC-silenced SKOV3-derived spheroids. Indeed, the results of miRNA TaqMan Assay analysis (Figure 7D) show that FHC knock down leads to a dramatic up-regulation of both miRNAs.

Taken together, these results suggest that ferritin may be controlling several aspects related to cancer cell proliferation, migration and stem cell expansion through a complex regulatory network involving several miRNAs.

### FHC silencing causes imbalance in the metabolism of unsaturated fatty acids and over-expression of stem cell markers in SKOV3-derived spheroids

**Figure 8:**
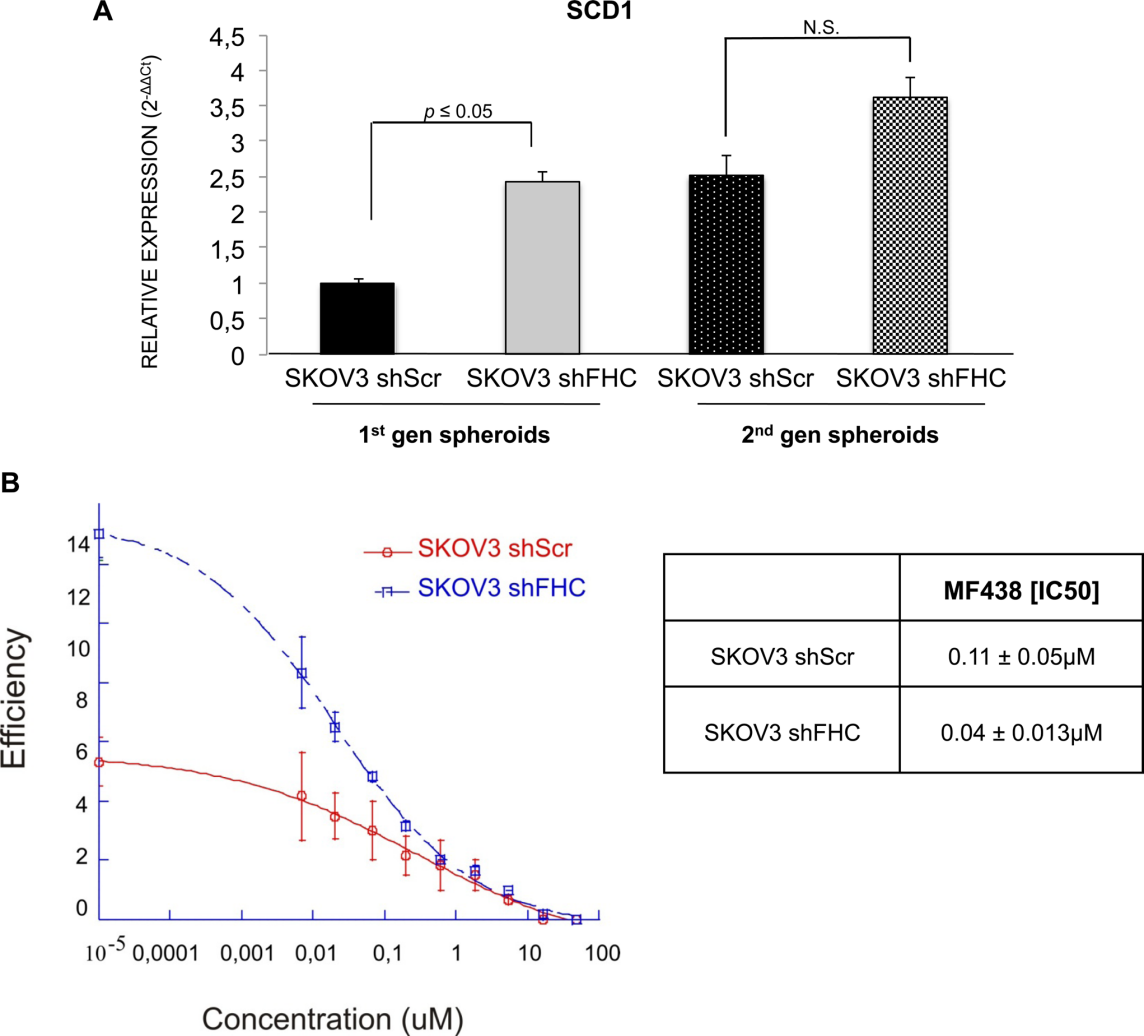
Stearoyl-CoA desaturase is up-regulated in shFHC SKOV3 spheroids SCD1 expression level, analyzed by qRT-PCR, is higher in shFHC spheroids than shScr SKOV3 spheroids. qRT-PCR results are shown as means +/− SD (**A**). The Curves represent ovarian cancer spheroids incubated in presence of different concentrations of MF438 for a 96 hours. The dose–response curves were defined with Kaleidagraph software. Results reflect the means +/− SD of 3 independent experiments (**B**).

A robust body of evidence in the literature suggests that lipid biosynthesis and desaturation are required for the survival of tumor cells. Stearoyl-CoA-desaturase (SCD1) is a key enzyme in unsaturated fatty acids metabolism, whose expression is related to cancer progression [39]. We have recently shown that SCD1 expression is up-regulated in lung cancer spheroids and that its inhibition either by siRNA silencing or by chemical compounds strongly affects cancer spheroid propagation and expression of stem cell markers [28]. Since SKOV3 cells silenced for FHC show an increased spheroid efficiency, we decided to assess whether this could be linked to increased SCD1 expression levels. SCD1 expression levels were similar in adherent cultures of SKOV3 shScr and SKOV3 shFHC cells (not shown). However, shFHC SKOV3-derived spheroids showed a significant increase of SCD1 mRNA levels, indicating that FHC might contribute to dysregulate directly or indirectly lipid pathways essential for the survival of cancer stem cells (Figure 8A). In order to evaluate the role of SCD1 in spheroid formation in SKOV3 cells, both FHC-silenced and control cells were subjected to spheroid forming assay in the presence of increasing concentrations of MF-438, a potent and selective small molecule inhibitor of the enzyme [28]. The results (Figure 8B) show a strong dose-dependent inhibition of spheroids in both cells. Interestingly, the sensitivity of shFHC-cells to MF-438, measured as IC_50_, was greater than shScr cells. This suggests a greater dependence of cells silenced for FHC to SCD1 activity.

## DISCUSSION

Ovarian cancer is the leading cause of mortality from gynecologic malignancies [40]. Despite modest improvements in response rates, progression-free survival and median survival using chemotherapy with adjuvant platinum and taxanes following surgery, overall survival remains very low [41]. This is mainly due to two reasons. Firstly, the majority of patients are diagnosed with advanced disease when cancer cells have already metastasized, mainly by diffusion through the peritoneal cavity followed by implantation on the mesothelial lining of the peritoneum [42]. Secondly, although patients initially respond to therapy, recurrence rates are very high, and once relapses occur, tumour cells have developed chemoresistance.

A heterogeneous mixture of cells are found in ovarian cancer tissue, including a subpopulation of CSCs responsible for tumor initiation, maintenance and relapse following chemotherapy. These cells exist in the form of multicellular 3D spheroids in malignant ascites of patients with advanced epithelial ovarian cancer [43]. Spheroids serve as vehicles for cancer cell dissemination, protecting cells from environmental stress-induced anoikis [44]. It has been previously shown that cells present in spheroids are enriched in a set of stem cell markers including ALDH1, CD133, CD117, Nanog and Oct4 [45, 48].

The present study stems from the observation that in patients with serous ovarian cancer at stage II–III, low FHC mRNA expression levels is associated with shorter survival. These data led us to hypothesize that high FHC expression may confers a less aggressive disease. The major finding of the present work is that silencing the expression of the ferritin heavy chain (FHC) results in up-regulation of the same set of markers indicated above as linked to ovarian cancer stemness together with enhanced spheroid formation, improved migratory ability and EMT.

A growing body of evidences indicate that ferritin is involved not only in the intracellular iron metabolism but also in many biochemical pathways related to cancer onset and progression. As recently reviewed by Min Pang and Connor [49], these pathways include, among others, control of cell proliferation, inhibition of cell death and apoptosis, induction of epithelial to mesenchymal transition. The specific role of ferritin in these intricate processes is far from being totally defined, also due to cell-specific differences that may lead to conflicting results. However, a survey of the literature indicates that the involvement of ferritin in cancer biology is related either to its role of iron scavenger or to iron-independent activities. Over-expression of FHC in HeLa cells, for instance, induces a consistent decrease (about 50%) of apoptosis [3]. Most importantly, the same inhibitory effect was obtained with a mutant FHC lacking the ferroxidase activity [50]. FHC physically binds to and activates p53, which, in turn, regulates at transcriptional level FHC expression [14]. Also this feedback loop is iron independent, since the binding of FHC to p53 is detached from its ferroxidase activity. In other cases the ferritin anti-oxidant properties seem to play a decisive role: this is the case of human mesothelioma cells, where ferritin reduces H_2_O_2_-induced apoptosis, or the case of the TGF-β-induced epithelial to mesenchymal transition in murine hepatocytes, where the down-regulation of FHC is accompanied by an increase in ROS production [51].

In the last years we have analyzed the FHC functions in human transformed cell lines by using the shRNA technology to down-regulate its amounts. FHC-silencing of a human metastatic melanoma cell line results in a substantial modification of gene expression that is accompanied, *in vitro*, by decreased growth activity and reduced invasiveness and cell adhesion capabilities, and, *in vivo*, by a 4-fold reduction of the tumor growth capacity [20]. The ability of FHC in modulating gene expression is not restricted to melanoma cells, as revealed by transcriptome analysis of FHC-silenced K562 cells [52]. In K562 cells we identified a repertoire of FHC-dependent miRNAs, which, in turn, affect RAF1/pERK1/2 expression leading to a reduced proliferation rate in the silenced cells [21].

Also in the case of the present work on ovarian cancer SKOV3 cells, we observe that FHC silencing results in altered expression of a network of miRNAs. Although we focus our analysis on a limited set of three specific microRNAs, namely miR-125b, miR-146a and miR-150, the results are very interesting. In first instance FHC silencing causes a potent up-regulation of miR-146a and miR-150 which were previously shown to directly control size and number of ovarian cancer spheroids, to stimulate cell survival and to increase drug resistance to cisplatin, all features linked to the presence of CSCs [38]. These two miRNAs are differentially up-regulated in omental lesions as compared to primary tumors and have been postulated to be responsible for the emergence of drug resistant disease. The other miRNA we find deregulated in the opposite direction is miR-125b. The miR-125 family is a highly conserved miRNA family composed of four members whose aberrant expression is tightly related to tumor development. miR-125 family members can act either as oncosuppressor or oncogenes depending on the cellular context [53, 54]. In SKOV3 cells miR-125b clearly behaves as an oncosuppressor and a downstream mediator of FHC activity. Not only its expression is decreased upon FHC gene silencing, but in addition its enforced expression in FHC silenced cells is able to revert several of the features of wild type SKOV3 cells.

In this paper we have uncovered a new function of FHC in the control of ovarian cancer cell proliferation, migration and EMT which is in part due to orchestration of a complex network of microRNAs. However, we cannot exclude that FHC control of ovarian cancer stemness is exerted at additional levels. Indeed, we have recently observed that FHC silenced SKOV3 cells present increased pAKT levels, most likely as a consequence of released interference with CXCR4 intracellular signaling (FC, manuscript in preparation). It is possible therefore that the phenotypic changes we observe in FHC-silenced ovarian cancer cells are the consequence of pleiotropic and still largely uncharacterized functions of this protein both at the nuclear and cytoplasmic levels.

The natural conclusion of our observations is that FHC exerts a negative control on CSCs formation and that fluctuations in its expression levels may, therefore, play a role in regulating the amount of CSCs generated within ovarian cancer, and therefore the formation of metastasis and relapse after chemotherapy. This finding has profound implications for our understanding of the biology of ovarian cancer and for the design of new therapies. In this regard, it will be interesting in the future to assess in a large number of ovarian cancer lesions whether FHC expression is linked with disease progression and metastasis and also whether it may behave as a prognostic factor.

## MATERIALS AND METHODS

### Cell cultures

SKOV3 human ovarian cancer cells (ATCC, Manassas, VA, USA) were cultured in two different conditions: i) for adherent cultures, cells were grown in RPMI 1640 (Sigma-Aldrich, St. Louis, MO, USA) medium supplemented with 10% fetal bovine serum (FBS) and 1% Penicillin-Streptomycin (Sigma-Aldrich); ii) for sphere cultures, cells were grown in DMEM F12 (Sigma-Aldrich) supplemented with B27 (Life Technologies, Carlsbad, California, USA), heparin, epidermal growth factor (EGF) and basic fibroblast growth factor (bFGF) (Sigma-Aldrich) (Sphere medium). In both conditions, cells were maintained at 37°C in a humidified 5% CO_2_ atmosphere. HEK-293T cells (Sigma-Aldrich) were cultured in adherent conditions in DMEM (Sigma-Aldrich) medium with 10% FBS and 1% Penicillin-Streptomycin at 37°C in a humidified 5% CO2 atmosphere.

MCF-7 human breast adenocarcinoma cell line (ATCC, Manassas, VA, USA) were cultured in spheroid conditions and maintained in sphere medium as described above at 37°C in a humidified 5% CO_2_ atmosphere.

### Preparation of lentiviral supernatants and transduction of SKOV3 cells

Lentiviral preparation and transduction were performed as described in Mega et al. [55]. Briefly, 5 × 10^6^ HEK-293T cells were grown on 10 cm plates to 70–80% confluence and co-transfected with 10 μg of shRNA lentiviral DNA, 2 μg of pCMV-VSV-G expressing envelope plasmid, and 18 μg of packaging viral CMV delta 8.9 plasmid, using the calcium phosphate precipitation method. After 8 hours, fresh medium was added, and cells were cultured for additional two days. After 48 h, the medium was harvested and filtered through 0.45 μm filters. The supernatants were used to cross-transduce SKOV3 cells in the presence of 8 μg/ml polybrene (Sigma-Aldrich) and positive clones were isolated by puromycin selection (1 μg/ml). Cells were stably transduced with a lentiviral DNA containing either an shRNA that targets the 196–210 region of the FHC mRNA (sh29432) (SKOV3 shFHC) or a control shRNA without significant homology to known human mRNAs (SKOV3 shScr).

### FHC transient silencing and reconstitution

SKOV3 shFHC cells were seeded in six-well plates at 3 × 10^5^ cells/well and grown overnight prior to transfection. All the transfection were performed by using Lipofectamine 2000 transfection reagent (Roche, Indianapolis, IN) following manufacturer's instructions. FHC reconstitution was performed using 1,4 μg/μl of the expression vector containing the full length human FHC cDNA (pc3/FHC) (SKOV3 shFHC^pc3FHC^) while 1,4 μg/μl of pcDNA3.1 plasmid was used as negative control (SKOV3 shFHC^pcDNA3.1^). FHC transient silencing was performed using a pre-cast small-interfering RNA siFHC and a siRNA *Silencer*^®^ Negative Control (Thermo Fisher Scientific, Massachusetts, USA). All transfection experiments were repeated twice.

### Spheroid-forming assays

For spheroid formation, adherent FHC-silenced and non-silenced SKOV3 cells were trypsinized and two different cell concentrations per well (1 × 10^3^/ml and 1 × 10^2^/ml) were seeded in ultra low attachment 6-well plates (Corning Incorporated, Corning, NY, USA), using sphere medium described above. After six days, the first generation spheroids were dissociated using 500 μl accumax (Millipore, Temecula, CA, USA) and then centrifuged at 200 × g for 5 min. Dissociated cells were resuspended in sphere medium and plated in second generation. The same procedure was performed to obtain all subsequent generations. Spheres were counted and photographed (magnification of 10×) using the Leica DFC420 C and Leica Application Suite Software.

### RNA extraction and semi-quantitative reverse transcriptase polymerase chain reaction (RT-PCR)

Total RNA was extracted from FHC-silenced SKOV3 (SKOV3 shFHC) and scrambled-shRNA SKOV3 (SKOV3 shScr) by using Trizol method according to the manufacturer's instructions (Life Technologies, Carlsbad, California, USA). For each culture type, two biological replicates were used. All the RNA samples were DNase-1 treated (Ambion, Austin, TX), and purity and integrity of the RNA was checked spectroscopically and by gel electrophoresis before use. Then, 1 μg of purified RNA was reverse-transcribed by using High Capacity cDNA Reverse Transcription kit (Life Technologies, Carlsbad, California, USA). Subsequently, 50 ng of cDNA of each sample was amplified with the following primers: FHC-F: 5′-catcaaccgccagatcaac-3′; FHC-R: 5′-gatggctttcacctgctc at-3′; GAPDH-F 5′-aacaccaccatggagaaggc-3′; GAPDH-R 5′-acagccttggcagcaccact-3′. Human GAPDH cDNA fragment was amplified as the internal control. The amplified products were electrophoresed on 1% agarose gels.

### Western blotting analysis

SKOV3 FHC-silenced and non-silenced cells were lysed in the following buffer [20 mM Hepes pH 7.9, 420 mM NaCl, 1% Triton X-100, 1 mM EDTA, 25% glycerol, 1 mM PMSF, 1 mM Na3VO_4_, 1 mM DTT, 1 μg/ml aprotinin, 1 μg/ml leupeptin] for 30 min on ice. After removing cell debris by centrifugation (12,000g × 30 min), the concentration of protein extracts was measured by the Bio-Rad protein assay according to the manufacturer's instructions (BioRad Laboratories). A total of 70 μg protein extract was boiled for 10 min in SDS sample buffer, separated by 12% SDS-PAGE and transferred to a nitrocellulose membrane by electroblotting. Non-specific reactivity was blocked in non fat dry milk in TPBS [5% (w/v) milk in PBS (pH 7.4) and 0.005% Tween 20] for 2 h at room temperature. The membrane was incubated with rabbit anti-H ferritin antibody (1:200; Santa Cruz Biotechnology, Texas, USA), rabbit anti-Vimentin antibody (1:500; Cell Signaling Technology, Danvers, MA, USA) and rabbit anti-TCF8/ZEB1 antibody (1:1000; Cell Signaling Technology, Danvers, MA, USA) over-night at 4°C, followed by incubation with goat anti-rabbit secondary antibody (1:5000; Santa Cruz Biotechnology). The membrane was developed by ECL-Western blot detection reagents according to the manufacturer›s instructions (Santa Cruz Biotechnology). γ-Tubulin was used as a loading control.

### Quantitative real-time PCR (qRT-PCR)

Gene expression analysis was assessed by real-time PCR using the cDNA obtained from the first and the second generation of SKOV3 shScr and SKOV3 shFHC spheroids, from SKOV3 shScr, SKOV3 shFHC, reconstituted SKOV3 cells (SKOV3shFHC^pc3FHC^). Primer sequences used for amplifications were as follows: GAPDH-F 5′-aacaccaccatggagaaggc-3′; GAPDH-R 5′-ac agccttggcagcaccact-3′; SCD1-F 5′-aaagctcggctgcccctacg-3′; SCD1-R 5′-ttgtggtgggcacggtgg tc-3′.

Stemness markers used for stemness analysis were Sox2, Oct4, Nanog, ALDH1 and primer sequences used for amplifications were: Sox2-F 5′-gtgcagaggaaaccgaagag-3′; Sox2-R 5′-tcattgctgcacaccttctc-3′; Oct4-F5′-catcaaccgc cagatcaac-3′; Oct4-R5′-gatggctttcacctgctcat-3′; Nanog-F 5′-aaccaaaggatgaagtgcaagcgg-3′; Nanog-R 5′-tccaagttggg ttggtccaagtct-3′; ALDH1-F 5′-ctgggctgacaagattcatggtc a-3′; ALDH1-R 5′-ggtgaagagccgtgagaggagttt-3′.

EMT markers were Vimentin and E-cadherin and primer sequences used were: Vim-F 5′-aggaaatg gctcgtcaccttcgtg-3′; Vim-R 5′-ggagtgtcggttgttaagaactag-3′; E-cadherin-F 5′-tacgccgggactccaccta-3′; E-cadherin-R 5′-ccagaaagcgaggcctgat-3′. qRT-PCR were performed as described in Biamonte et al. [21]. Every experiment was performed in triplicate.

### Immunofluorescence

For immunofluorescence analysis, cells were treated as previously described by Zolea et al. [56]. Primary antibody (ferritin heavy chain H-53, 1:100 Santa Cruz Biotechnology) was diluted in blocking buffer and incubated for 1h at 37°C. To visualize binding, an appropriate secondary antibody (Alexa Fluor 488, 1:400, Invitrogen, Carlsbad, CA) diluted in blocking buffer was applied for 30 min at 37°C. Nuclear Dapi (1:1000, Invitrogen, Carlsbad, CA) was added for 20 min after secondary antibody incubation, prior to washing and mounting (ProLong Gold antifade Reagent, Molecular Probes, Eugene, OR). Images were collected using a Leica TCS SP2 confocal microscopy system (63× objective).

### Chemical compound and treatment

2-methyl-5-(6-(4-(2-(trifluoromethyl)phenoxy)piperidin-1-yl)pyridazin-3-yl)-1,3,4-thiadiazole (MF-438) was synthesized as previously described [57], dissolved in DMSO and stored at −20°C. For each experiment, the stock solutions were further diluted in medium to obtain the desired concentrations. The final solvent concentration in cell culture was ≤ 0.1% v/v of DMSO. Parallel cultures of cells in medium with DMSO were used as controls.

### Cell proliferation assays

Cell proliferation was tested using a 3-(4,5-dimethylthiazol-2-yl)-2, 5-diphenyl tetrazolium bromide (MTT) (Sigma-Aldrich) assay as described by Biamonte et al. [21].

### Glucose assay

1 × 10^5^ cells from adherent cell cultures were seeded in 10 ml of RPMI1640 (Sigma-Aldrich), supplemented with FBS and Penicillin-Streptomycin (Sigma-Aldrich) in 100 mm plates. After 24, 48 and 72 hours, 500 μl of supernatant were taken and glucose concentration was measured using COBAS 6000 instrument (Roche, Indianapolis, IN). The assay was performed three times.

### Wound healing assays

Wound healing assays were used to evaluate *in vitro* cell migration capacity. SKOV3 shScr, SKOV3 shFHC, SKOV3 shFHC^pc3FHC^, SKOV3 siFHC and SKOV3 cntr cells were seeded in a 6-well plate and cultured until reaching *confluence.* For simulating a wound, a pipette tip was used to make a scratch. After 4, 6, 12, 17, 20 and 24 hours, cells were monitored and images of wound healing were captured (magnification of 10×) using the Leica DFC420C and Leica Application Suite Software. Subsequently, cell migration was quantified by measuring the migration distance with ImageJ 64 software.

### miRNA transfections and expression assays

SKOV3 shFHC cells were seeded at 5 × 10^5^ cells/well in a 6-well plate (Corning Incorporated, Corning, NY, USA). After 24 h, cells were transfected with 100 nM of hsa-miR-125b-5p mirVana miRNA mimic (Life Technologies, Carlsbad, California, USA) and with 100 nM of mirVana mimic negative control #1 using 6 μl of Lipofectamine 2000 reagent (Life Technologies, Carlsbad, California, USA) per sample, according to the manufacturer's instructions. After 6 h of incubation, the transfection medium was replaced with fresh RPMI1640 medium. Then, after 48 h of transfection, cells were centrifuged and total RNA was extracted from both SKOV3 shFHC transfected with hsa-miR-125b-5p mimic and negative control.

miRNA reverse transcription was performed using 10 ng of RNA from SKOV3 shScr and SKOV3 shFHC, either in adherent or spheroid conditions, 3 μl of TaqMan MicroRNA Assay (5×) and High Capacity cDNA reverse transcription kit (Life Technologies, Carlsbad, California, USA). The thermal profile consisted of 3 steps: 25°C for 10 min, 37°C for 120 min, 85°C for 5 min. The analysis of miRNAs expression profile was performed by miRNA TaqMan Assay method as described by Biamonte et al. [21]. RNU-U6 was used as housekeeping. Each experiment was performed in triplicate.

### Analysis of surface stem cell markers in shScr and shFHC SKOV3 spheroids

Spheres obtained from FHC-silenced and non-silenced SKOV3 cells were dissociated with Accumax (Millipore, Temecula, CA, USA) and centrifuged at 300 × g for 5 minutes at 4°C. Antibodies conjugated to a fluorescent tag, specific for CD117, CD133, CD34 (BD Biosciences), were added to the cell pellet and incubated for 30 minutes on ice. Post-incubation, cells were washed with 10 mL of PBS 1X and centrifuged at 300 × g for 5 minutes at 4°C. Samples were analysed using a FACScan (BD Biosciences).

### Statistical analysis

All experiments were conducted at least two times and each result is expressed as mean values +/−standard deviation. The comparisons between means of different groups were performed using Student ‘s *t*-test and a *p*-value less than 0.05 was considered statistically significant.

### Bioinformatic analysis

Meta-analyses of published ovarian cancer microarray datasets were performed by using online tool (www.kmplot.com/ovar). The FTH1 gene expression data of 723 ovarian cancer patients were downloaded from Gene Expression Omnibus and The Cancer Genome Atlas [32].

## SUPPLEMENTARY MATERIALS FIGURES


