# Supplementary material for: Ferritin heavy chain is a negative regulator of ovarian cancer stem cell expansion and epithelial to mesenchymal transition
Source: Oncotarget. 2016 Aug 22;7(38):62019–33. doi: 10.18632/oncotarget.11495 (PMC5308708; doi:10.18632/oncotarget.11495)
Supplement: Supplementary file 1 [file oncotarget-07-62019-s001.pdf]

Ferritin heavy chain is a negative regulator of ovarian cancer stem cell expansion and epithelial to mesenchymal transition

Supplementary Materials

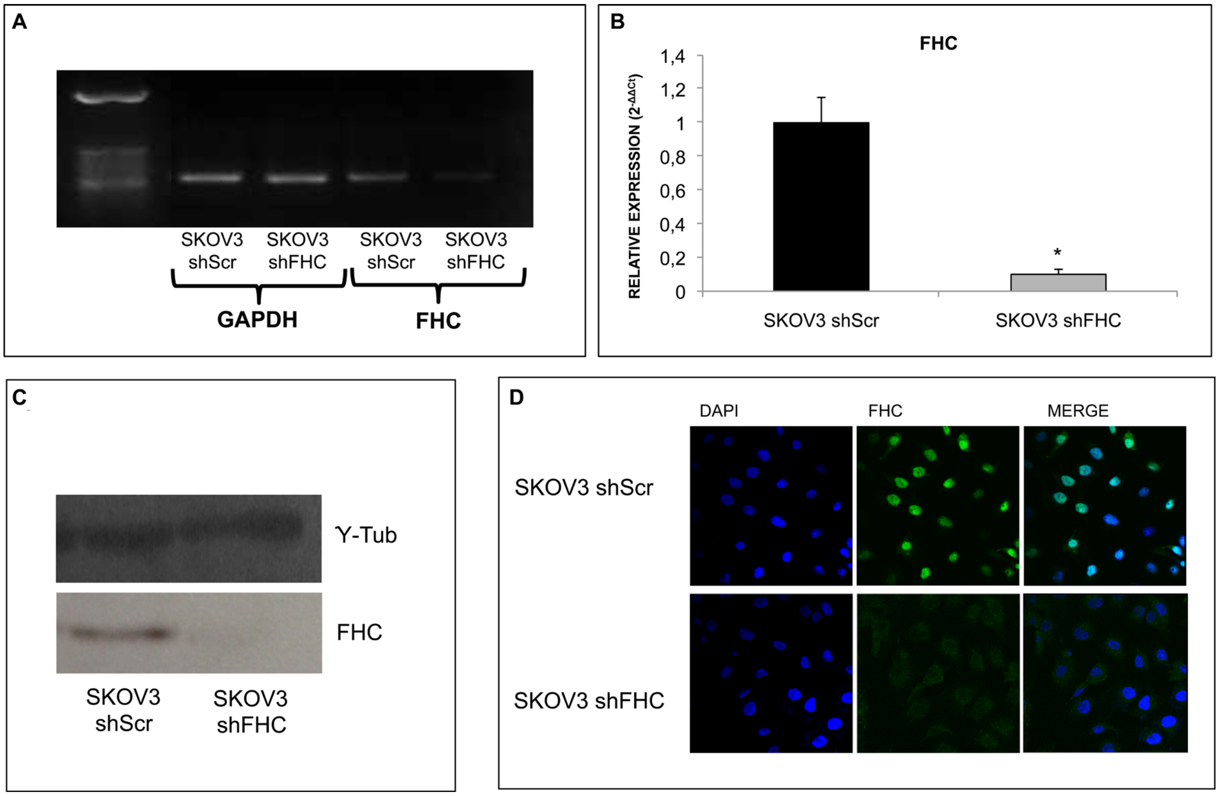

Supplementary Figure S1: FHC-silencing in SKOV3 cells. FHC mRNA expression levels in shScr and shFHC SKOV3 cells measured by PCR (**A**) and qPCR (**B**). FHC protein amounts measured by western blot analysis (**C**) and IF (**D**). *p value ≤ 0.05.

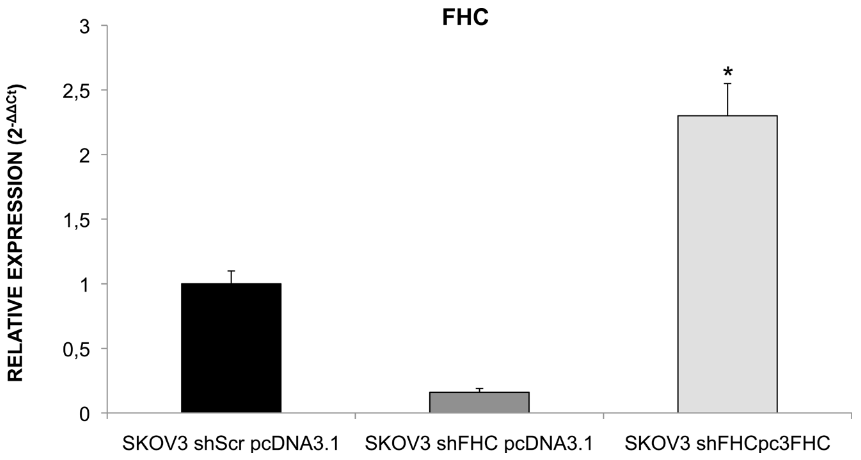

Supplementary Figure S2: FHC-reconstitution in SKOV3 shFHC cells. FHC mRNA expression levels in FHC-silenced cells measured by qPCR after FHC transient reconstitution using a vector containing the full length human FHC cDNA (SKOV3 shFHC^{pc3FHC}). *p value ≤ 0.05 compared to SKOV3 shFHC pcDNA 3.1.

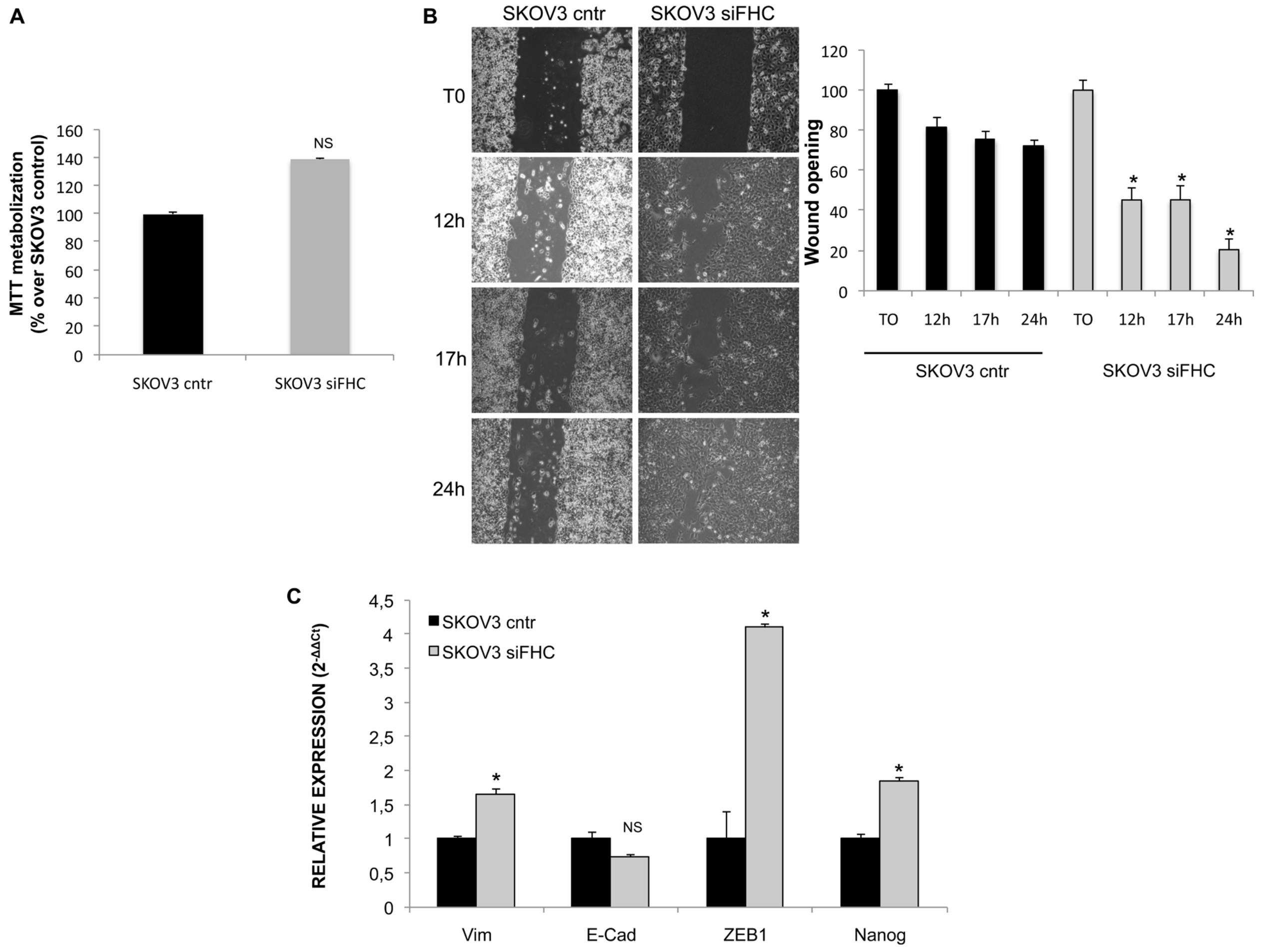

Supplementary Figure S3: FHC transient silencing mimic the more tumorigenic phenotype of FHC stable silencing in SKOV3 cells. Cell proliferation and viability, measured by MTT assay, is higher in FHC-transiently silenced (SKOV3 siFHC) compared to control (SKOV3 cntr) SKOV3 cells at 48h albeit without reaching a statistical significance. The experiment was performed in duplicate and data are shown as mean +/− SD (**A**). SKOV3 siFHC cells exhibit a significantly higher migration capacity compared to SKOV3 cntr cells at 12, 17 and 24 h. Wound size was quantified by ImageJ 64 software. Images and quantification are representative of two different experiments and the values are given as mean +/− SD. *p value ≤ 0.05 (**B**). FHC-transient silencing significantly promotes mesenchymal Vimentin and ZEB-1 markers while inhibits E-cad without statistical significance. mRNA expression of NANOG stemness markers is significantly increased in SKOV3 siFHC compared to control SKOV3 cells. qRT-PCR results are shown as means +/− SD. *p value ≤ 0.05 (**C**).

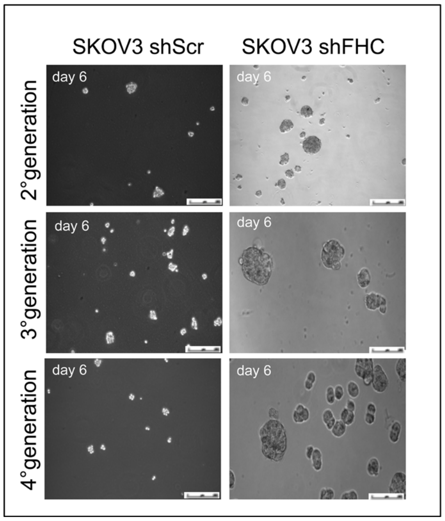

	SKOV3 shScr 100cells/well	SKOV3 shFHC 100cells/well
2nd generation		
day 6	4	15
3rd generation		
day 6	3	14
4th generation		
day 6	-	15

Supplementary Figure S4: Sphere-forming assay at lower cell density. SKOV3 shScr and SKOV3 shFHC cells were cultured in low-attachment conditions at very low cell density/well (100 cells/well) and formation of spheroids was observed. Photographs of representative spheroids from each cell type and a table with the tumour spheroid number as a function of culture time, are reported. FHC-silencing induced an increase in spheroids number and size at each generation. Results are representative of two different experiments.

A

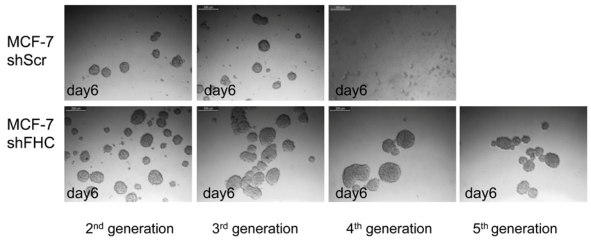

	MCF-7 shScr 1000cells/ well	MCF-7 shFHC 1000cells/well
2nd generation		
day 6	16	28
3rd generation		
day 6	10	18
4th generation		
day 6	7	15
5th generation		
day 6	-	11

B

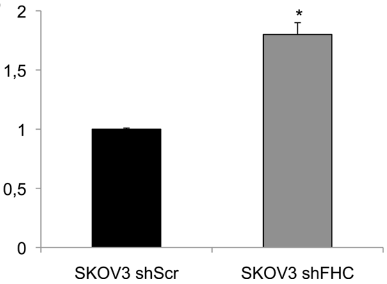

Supplementary Figure S5: Comparison of sphere-forming ability between FHC-silenced and non-silenced MCF7 cells. FHC-silencing induces an increased number and size of spheroids in MCF-7 cells. Starting from an equal quantity of cultured cells per well, the rate of sphere formation of every single generation in MCF-7 shScr is slower than that in MCF7 shFHC cells. (**A**). FHC-silencing up-regulates the expression of the stem cell biomarker OCT4. The qRT-PCR analysis was performed in duplicate and data are shown as mean +/− SD. **p* value ≤ 0.05 (**B**).

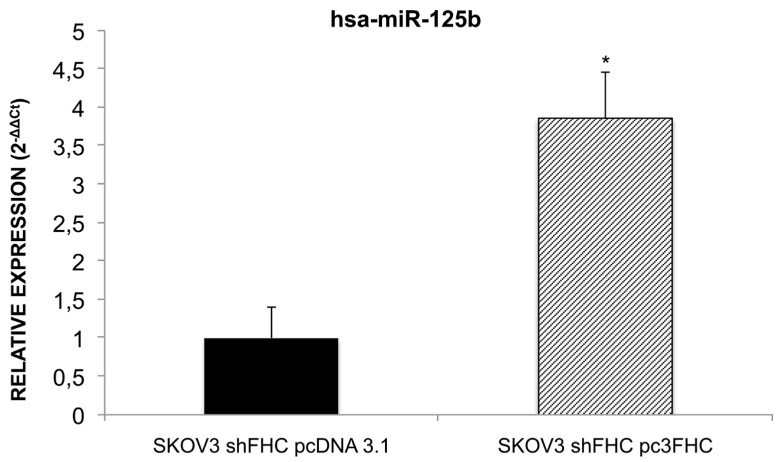

Supplementary Figure S6: miR-125b expression is up-regulated after FHC reconstitution of SKOV3 FHC-silenced cells. miR-125b expression, measured by TaqMan Assay, is positively regulated by FHC expression rescue of SKOV3 shFHC cells. The result is representative of two different experiments and data are reported as mean +/− SD. **p* value ≤ 0.05.
